# 肺鳞癌的免疫治疗进展

**DOI:** 10.3779/j.issn.1009-3419.2016.10.09

**Published:** 2016-10-20

**Authors:** 守正 王, 峻岭 李

**Affiliations:** 100021 北京，国家癌症中心/中国医学科学院北京协和医学院肿瘤医院 National Cancer Center/Cancer Hospital, Chinese Academy of Medical Sciences and Peking Union Medical College, Beijing 100021, China

**Keywords:** 肺肿瘤, 免疫治疗, 免疫检查点抑制剂, 肿瘤疫苗, Lung neoplasms, Immunotherapy, Checkpoint inhibitors, Tumour vaccines

## Abstract

近几年来，肺鳞癌在化疗及靶向治疗上的进展不够显著，但免疫治疗却在肺鳞癌的治疗上取得了突破性的进展。免疫治疗通过免疫系统来清除肿瘤细胞，主要分为免疫检查点抑制剂及治疗性疫苗。免疫检查点抑制剂，包括抗细胞毒性T淋巴细胞抗原4（cytotoxic T-lymphocyte associated antigen 4, CTLA-4）抗体与抗程序性死亡受体-1（programmed death receptor 1, PD-1）抗体等多种药物已进行了肺鳞癌的Ⅱ期、Ⅲ期临床试验，并取得了一定成果。免疫治疗将成为肺鳞癌治疗的一种重要手段。

## Abstract (en)

In recent years, squamous non-small cell lung cancer (NSCLC) didn't progress much in chemotherapy or target therapy. However, immunotherapy has made breakthroughs in treating squamous NSCLC. Immunotherapy includes two main broad classes of immune checkpoint inhibitors and therapeutic vaccines. Immune checkpoint inhibitors, including anti cytotoxic T-lymphocyte associated antigen 4 (CTLA-4) and anti programmed death receptor 1 (PD-1) antibodies, have been tested in the phase Ⅱ/Ⅲ clinical trials and have demonstrated promising outcomes. Immunotherapy will become an important treatment for squamous NSCLC.

肺癌在中国^[1]^乃至全球^[2]^范围内均为癌症相关死亡的主要致死原因之一，其中85%的新发患者诊断为非小细胞肺癌（non-small cell lung cancer, NSCLC），而又有约30%的患者为肺鳞癌^[3]^。大多数患者在确诊肺癌时已处于晚期（Ⅲb期/Ⅳ期），含铂的两药联合化疗则成为晚期NSCLC的主流治疗方案。此后，针对表皮生长因子受体（epidermal growth factor receptor, *EGFR*）基因突变及间变性淋巴瘤激酶（anaplastic lymphoma kinase, *ALK*）基因重排的患者，又展开了针对EFGR及ALK靶点的靶向治疗。但是，只有相对较少的一部分患者可以从靶向治疗中获益，且多为腺癌患者。针对肺鳞癌治疗的进展却很少，这可能与肺鳞癌患者有着更高的突变率和固有耐药性有关。然而，随着免疫治疗的不断发展，为肺鳞癌的治疗打开了新的大门。本文就目前肺鳞癌的免疫治疗相关进展进行综述。

## 1 免疫检查点抑制剂

### 1.1 抗细胞毒性T淋巴细胞抗原4（cytotoxic T-lymphocyte associated antigen 4, CTLA-4）

CTLA-4又称为CD152，是一种由*CTLA*-*4*基因编码的跨膜蛋白。其可以表达在活化的效应CD4^+^、CD8^+^ T细胞以及调节T细胞上，与CD28竞争性地结合抗原提呈细胞表面的共刺激分子B7-1及B7-2，且CTLA-4与B7分子的亲和力要远高于CD28^[4, 5]^。CD28与B7分子结合后，通过产生IL-2和抗细胞凋亡因子来促进T细胞增生^[6]^。与CD28相反，CTLA-4为负性调节因子。CTLA-4被激活后，会减弱下游的由T细胞受体与CD28诱导的激酶信号，如PI3K/AKT通路，从而抑制T细胞^[7-10]^。由于B7-1、B7-2表达在抗原提呈细胞表面，CTLA-4抑制抗肿瘤免疫的作用被认为是在T细胞活化的次级淋巴器官中产生，而不是在肿瘤微环境中^[11]^。

依匹木单抗（Ipilimumab）是一种全人源化的IgG1抗CTLA-4单克隆抗体，他可以阻断CTLA-4与配体结合，进而增强抗肿瘤的免疫应答。基于其在Ⅲ期临床试验中显示出的总生存期（overall survival, OS）的获益，依匹木单抗成为第一个被批准用来治疗无法手术或晚期黑色素瘤的免疫检查点抑制剂^[12, 13]^。在一项依匹木单抗联合化疗治疗晚期NSCLC的Ⅱ期临床试验中^[14]^，204例既往未接受过化疗的患者按1:1:1的比例随机分为3组，分别接受紫杉醇+卡铂+同步依匹木单抗（前4周期依匹木单抗，后2周期安慰剂）、紫杉醇+卡铂+序贯依匹木单抗（前2周期安慰剂，后4周期依匹木单抗）以及紫杉醇+卡铂+安慰剂。对于未进展且能耐受进一步治疗的患者会继续接受每12个月一次的依匹木单抗或安慰剂的维持治疗。与对照组相比，序贯依匹木单抗组达到了改善免疫相关无进展生存期（immune-related progression-free survival, irPFS）的研究终点（5.7个月*vs*4.6个月，HR=0.72，*P*=0.05）。序贯依匹木单抗疗法还提高了修订版世界卫生组织（World Health Organization, WHO）标准的PFS（mWHO-PFS）（HR=0.69, *P*=0.02）。尤其是鳞癌的患者，接受序贯依匹木单抗治疗后的irPFS与mWHO-PFS改善更为明显。鳞癌与非鳞癌患者免疫相关性无疾病生存期（immune-related PFS, irPFS）的HR分别为0.55（95%CI: 0.27-1.12）及0.82（95%CI: 0.52-1.28），mWHO-PFS的HR分别为0.40（95%CI: 0.18-0.87）及0.81（95%CI: 0.53-1.26）。在鳞癌患者亚组中，序贯依匹木单抗组与对照组OS的HR=0.48（95%CI: 0.22-1.03），而在非鳞癌患者亚组中则为1.17（95%CI: 0.74-1.86）。

### 1.2 抗程序性死亡受体-1抗体

程序性死亡受体-1（programmed death receptor 1, PD-1），又称为CD279，是一种在外周组织中调节T细胞活性的Ⅰ型跨膜受体，表达于CD4^+^和CD8^+^淋巴细胞、B淋巴细胞和自然杀伤（natural killer, NK）细胞等细胞表面。PD-1包含免疫受体酪氨酸抑制模体及免疫受体酪氨酸转换模体。通过这两种模体，PD-1可以结合抑制性磷酸酶SHP2。此外，PD-1还参与激活PP2A，并直接抑制T细胞受体介导的效应功能，增加T细胞在组织内的迁移，从而限制了T细胞搜寻相互作用的细胞表面同源的多肽-主要组织相容性复合物的时间。T细胞可能因此忽略一些表达低水平多肽-主要组织相容性复合物的靶细胞^[10, 15]^。

PD-1主要有两种配体：PD-L1和PD-L2。PD-L1主要表达在实体肿瘤，PD-L2则高表达于B淋巴细胞的某些特定亚群^[16]^。T细胞一经抗原识别活化后，就会在其表面表达PD-1，并产生干扰素诱导多个组织中PD-L1的表达。PD-1与其配体结合后会抑制T细胞活性。因此，在正常情况下，PD-1/PD-L1通路通过负性调节免疫应答阻止自身抗体的过度刺激，维持对自身抗体的免疫耐受^[17]^。然而，PD-L1在肺癌中经常会过表达，导致肿瘤微环境中的免疫应答障碍^[18]^。PD-1/PD-L1的相互作用会抑制T淋巴细胞增殖、细胞因子释放及细胞毒性，导致肿瘤特异性T细胞衰竭与凋亡^[19]^。而阻断PD-1/PD-L1间相互作用则可以逆转衰竭的T细胞表型，并使抗肿瘤应答正常化^[20]^。

#### 1.2.1 纳武单抗（Nivolumab）

纳武单抗是一种全人源化IgG4单克隆PD-1抗体，与PD-1有着高亲和力，可以同时阻断PD-L1和PD-L2与PD-1结合^[21]^。是第一个被批准为用于化疗后晚期肺鳞癌患者的免疫检查点抑制剂。在Ⅰ期临床试验^[22]^中，所有剂量组总客观缓解率（objective response rate, ORR）为16.7%，中位缓解时间尚未达到（范围：3.7个月-36.8个月），中位OS为9.2个月（95%CI: 7.3-12.5），1年、2年、3年的OS分别为41%、24%、19%。

在一项纳武单抗治疗晚期、难治性肺鳞癌患者的多国Ⅱ期单臂临床试验（CheckMate 063）^[23]^中，ORR为14.5%，中位反应时间为3.3个月（四分位数间距：2.2-4.8），中位缓解时间在统计时尚未达到。25.6%的患者达到了疾病稳定（stable disease, SD），中位SD持续时间为6.0个月（95%CI: 4.7-10.9）。中位PFS及OS分别为1.9个月（95%CI: 1.8-3.2）与8.2个月（95%CI: 6.1-10.9）。6个月及1年PFS分别为25.9%与20.0%，1年OS为40.8%。

在一项随机双盲多中心的Ⅲ期临床研究（CheckMate 017）^[24]^中，272例既往接受过含铂方案化疗的晚期肺鳞癌患者被随机分为两组，分别接受纳武单抗或多西他赛治疗。纳武单抗与多西他赛组的反应率分别为20%与9%（*P*=0.008），中位OS分别为9.2个月（95%CI: 7.3-13.3）与6.0个月（95%CI: 5.1-7.3），1年OS分别为42%与24%，中位PFS分别为3.5个月与2.8个月（死亡或疾病进展的HR=0.62，95%CI: 0.47-0.81，*P*＜0.001）。纳武单抗组的死亡率为41%，明显低于多西他赛组（HR=0.59, 95%CI: 0.44-0.79, *P*＜0.001）。

#### 1.2.2 派姆单抗（Pembrolizumab）

派姆单抗也是一种人源化的IgG4单克隆PD-1抗体。在一项Ⅰ期临床试验（KEYNOTE-001）^[25]^中，495例晚期NSCLC患者随机接受每3周2 mg/kg或10 mg/kg派姆单抗或者每2周10 mg/kg派姆单抗。所有患者ORR为19.4%，中位缓解时间为12.5个月（范围：1.0-23.3），中位PFS为3.7个月（95%CI: 2.9-4.1），中位OS为12.0个月（95%CI: 9.3-14.7）。其中，鳞癌患者的ORR为23.5%，要明显优于非鳞癌患者（18.7%）^[25, 26]^。另一项在24个国家开展的多中心Ⅱ期/Ⅲ期临床试验（KEYNOTE-010）^[27]^中，1, 034例既往接受过治疗且表达PD-L1的晚期NSCLC患者按1:1:1的比例随机被分为三组，分别接受2 mg/kg派姆单抗、10 mg/kg派姆单抗或者多西他赛治疗，3组患者的OS分别为10.4个月（95%CI: 9.4-11.9）、12.7个月（95%CI: 10.0-17.3）及8.5个月（95%CI: 7.5-9.8）。与多西他赛组相比，低剂量与高剂量派姆单抗组的OS均有显著提高，HR分别为0.71（95%CI: 0.58-0.88, *P*=0.000, 8）与0.61（95%CI: 0.49-0.75, *P*＜0.000, 1）。对于肺鳞癌患者亚组，与多西他赛组相比，所有接受派姆单抗治疗的患者OS（HR=0.74; 95%CI: 0.50-1.09）与PFS（HR=0.86, 95%CI: 0.62-1.20）均能获益。结果不够显著的原因可能与样本量较少有关（222/1, 034）。此外，还有一些针对派姆单抗用于一线治疗NSCLC以及派姆单抗单药治疗无转移的NSCLC的临床试验正在开展中^[26]^。

#### 1.2.3 Atezolizumab

Atezolizumab是一种经设计的人源化的IgG1单克隆抗PD-L1抗体。Atezolizumab被设计成不会与Fc受体结合，从而阻断Fc效应功能。这一修饰消除了抗体依赖细胞介导的细胞毒性，进而避免可能存在的表达PD-L1的效应T细胞的损失及其抗肿瘤免疫的减弱。一项多中心开放的Ⅱ期随机对照试验（POPLAR）^[28]^中，287例既往接受过含铂化疗方案治疗后进展的NSCLC患者按1:1被随机分为2组，分别接受Atezolizumab或多西他赛治疗。Atezolizumab组与多西他赛组OS分别为12.6个月（95%CI: 9.7-16.4）与9.7个月（95%CI: 8.6-12.0），HR为0.73（95%CI: 0.53-0.99, *P*=0.04）。在鳞癌患者中，Atezolizumab组的总生存期要优于多西他赛组，OS分别为10.1个月与8.6个月（HR=0.80, 95%CI: 0.49-1.30）。

## 2 抗肿瘤疫苗

治疗性疫苗主要用来强化免疫系统，增强细胞毒性T细胞对于癌细胞表面特异性肿瘤相关抗原的反应。各种不同的癌症疫苗均有发展，可以用于不同来源的肿瘤抗原，以激活体液和细胞抗肿瘤免疫应答，包括全肿瘤细胞、DNA载体或细胞的特定部分，如蛋白质或肽^[29]^。一些基于抗原或全细胞的疫苗已进入晚期NSCLC的临床试验。

### 2.1 Tecemotide

粘蛋白1（mucin 1, MUC1）糖蛋白会在NSCLC细胞表面过度表达并异常糖基化^[30, 31]^。癌症相关MUC1与受体酪氨酸激酶或其他细胞表面受体异常作用。这些异常作用启动细胞内信号通路的不适当激活，促进了癌细胞的生长、增殖与生存^[30, 32-36]^。Tecemotide（L-BLP25）是一种MUC1抗原特异性疫苗，能够诱导T细胞对MUC1转基因的肺癌小鼠模型^[37]^和患者^[38-40]^的MUC1应答。

一项随机双盲Ⅲ期临床试验（START）^[41]^纳入了1, 513例既往接受过放化疗并达到疾病稳定（stable disease, SD）或有反应的Ⅲ期NSCLC患者，用以研究Tecemotide作为维持治疗的疗效。最终Tecemotide组及安慰剂组分别有829例与410例被纳入统计。Tecemotide组与安慰剂组的中位OS分别为25.6个月（95%CI: 22.5-29.2）与22.3个月（95%CI: 19.6-25.5），HR为0.88（95%CI: 0.75-1.03, *P*=0.123）。该试验中共纳入了572例肺鳞癌患者，Tecemotide组401例，安慰剂组171例。在既往接受同步放化疗的患者中，Tecemotide组和安慰剂组肺鳞癌患者的中位OS分别为28.0个月与19.6个月（HR=0.80, 95%CI: 0.59-1.09, *P*=0.155），无明显的统计学差异。同样，在序贯接受放化疗的患者中，Tecemotide组和安慰剂组肺鳞癌患者的中位OS分别为19.8个月与17.9个月（HR=0.88, 95%CI: 0.61-1.26, *P*=0.480），亦无明显的统计学差异。

### 2.2 黑色素瘤相关抗原3（melanoma-associated antigen 3, MAGE-A3）疫苗

MAGE-A3是一种几乎只有恶性细胞表达的蛋白，在NSCLC及其他实体瘤中均有表达^[42]^。MAGE-A3疫苗是一种全蛋白疫苗，包括重组融合蛋白（MAGE-A3和流感嗜血杆菌蛋白D）以及辅助增强免疫应答的ASO2B。在一项随机、双盲、安慰剂对照的Ⅲ期临床试验^[43]^中，2, 312例NSCLC患者被分为MAGE-A3组与安慰剂组，两组的PFS分别为60.5个月（95%CI：52.7-未达到）与57.9个月（95%CI：55.7-未达到）（HR=1.02, 95%CI: 0.89-1.18, *P*=0.74）。肺鳞癌亚组中MAGE-A3组与安慰剂组PFS的HR为0.98（95%CI: 0.79-1.21）。因该试验未得到MAGE-A3作为辅助治疗优于安慰剂的结果，目前已终止MAGE-A3治疗NSCLC的研究。

### 2.3 Belagenpumatucel-L

Belagenpumatucel-L是一种同种异体的全肿瘤细胞疫苗，包括人转化生长因子β2反义载体转染的4种NSCLC细胞系（H460、H520、SKLU-1以及RH2）。TGFβ主要在肿瘤微环境中发挥多种免疫抑制作用。下调TGFβ的表达可以增强疫苗的免疫原性以及对宿主NSCLC细胞的免疫应答。在一项随机、双盲、安慰剂对照的Ⅲ期临床试验^[44]^中，532例晚期NSCLC患者被随机分为Belagenpumatucel-L与安慰剂组，用以研究Belagenpumatucel-L作为NSCLC含铂化疗后维持治疗的疗效。Belagenpumatucel-L组与安慰剂组的中位OS分别为20.3个月（95%CI: 16.8-23.7）与17.8个月（95%CI: 13.7-22.0）（HR=0.94, 95%CI: 0.73-1.20, *P*=0.594）。两组的中位PFS分别为4.3个月（95%CI: 3.4-5.2）与4.0个月（95%CI: 3.0-5.0）（HR=0.99, 95%CI: 0.82-1.20, *P*=0.947）。在肺鳞癌患者亚群中，Belagenpumatucel-L组与安慰剂组的中位OS分别17.7个月和15.2个月，HR为0.92（95%CI: 0.58-1.46）。

## 3 总结与展望

免疫检查点抑制剂的相关研究进展为肺鳞癌的治疗翻开了新的篇章，但同时也有更多的领域需要去探索。目前主要以表达PD-L1作为可使用PD-1/PD-L1抑制剂患者的筛选标准，还有更多更精准的预测及预后因子急需被发现。而对于抗肿瘤疫苗来说，尽管目前的研究结果不尽如人意，但抗肿瘤疫苗与免疫检查点抑制剂联合使用也许可以互相增加疗效，值得进一步研究。虽然还有许多尚待研究的方面，但相信免疫治疗会给肺鳞癌患者带来新的福音。
